# Comparison of Retinal Thickness Measurements between the Topcon Algorithm and a Graph-Based Algorithm in Normal and Glaucoma Eyes

**DOI:** 10.1371/journal.pone.0128925

**Published:** 2015-06-04

**Authors:** Enting Gao, Binyao Chen, Jianling Yang, Fei Shi, Weifang Zhu, Dehui Xiang, Haoyu Chen, Mingzhi Zhang, Xinjian Chen

**Affiliations:** 1 School of Electronic and Information Engineering, Soochow University, Suzhou, China; 2 School of Electronic and Information Engineering, Suzhou University of Science and Technology, Suzhou, China; 3 Joint Shantou International Eye Center, Shantou University and the Chinese University of Hong Kong, Shantou, China; Duke University, UNITED STATES

## Abstract

**Purpose:**

To assess the correlation and agreement between the Topcon built-in algorithm and our graph-based algorithm in measuring the total and regional macular thickness for normal and glaucoma subjects.

**Methods:**

A total of 228 normal eyes and 93 glaucomatous eyes were enrolled in our study. All patients underwent comprehensive ophthalmic examination and Topcon 3D-OCT 2000 scan. One eye was randomly selected for each subject. The thickness of each layer and the total and regional macular thickness on an Early Treatment of Diabetic Retinopathy Study (ETDRS) chart were measured using the Topcon algorithm and our three-dimensional graph-based algorithm. Correlation and agreement analyses between these two algorithms were performed.

**Results:**

Our graph search algorithm exhibited a strong correlation with Topcon algorithm. The macular GCC thickness values for normal and glaucoma subjects ranged from 0.86 to 0.91 and from 0.78 to 0.90, and the regional macular thickness values ranged from 0.79 to 0.96 and 0.70 to 0.95, respectively. Small differences were observed between the Topcon algorithm and our graph-based algorithm. The span of 95% limits of agreement of macular GCC thickness was less than 28 μm in both normal and glaucoma subjects, respectively. These limits of total and regional macular thickness were 15.5 μm and 23.1 μm for normal subjects and 29.1 μm and 46.4 μm for glaucoma subjects, respectively.

**Conclusion:**

Our graph-based algorithm exhibited a high degree of agreement with the Topcon algorithm with respect to thickness measurements in normal and glaucoma subjects. Moreover, our graph-based algorithm can segment the retina into more layers than the Topcon algorithm does.

## Introduction

In glaucoma patients, the retinal nerve fibers are gradually damaged and lost, leading to thinning of the retinal nerve fiber layer (RNFL)[1–4]. Optic coherence tomography (OCT) is frequently used to measure the structural parameters of the optic nerve head (ONH) and the retinal RNFL thickness to evaluate glaucoma [2, 5, 6]. In recent years, an increasing number of researchers have utilized the thickness of the ganglion cell layer plus the inner plexiform layer (GCL+IPL)[7–9] as well as the ganglion cell complex (GCC, RNFL+GCL+IPL)[10, 11] of the macula to detect glaucoma. Therefore, it is very important for the ophthalmologist to have a reliable and efficient method to quantitatively analyze the retinal structural parameters.

OCT is the most commonly used imaging technology for macula examination. In particular, the recently developed spectral domain OCT (SD-OCT) can provides a non-invasive, in vivo, high-speed and high-resolution three-dimensions imaging of anterior and posterior eye structures.[12, 13]

Most commercially available SD-OCT cannot measure the thickness of the every retinal layers. For example, the Topcon 3D-OCT 2000 can only calculate the thickness of the following retinal layers: RNFL, GCL+IPL, IS/OS and RPE. Therefore, the discriminating ability of the Topcon 3D-OCT 2000 is limited. We have developed an accurate and reliable 3-D measurement of the thickness of all 10 retinal layers using macula-centered SD-OCT.

Although a few published studies have compared the macula layer thickness measurements between different software, most studies have focused on comparisons between commercial software[14–17], and some studies have been limited to only normal subjects[15, 18–20].

In this study, we compared the commercially available Topcon built-in algorithm and our graph-based algorithm by comparing the retinal ganglion cell complex (GCC, RNFL+GCL+IPL) thickness and retinal thickness of 9 sectors of ETDRS measurements for normal and glaucoma subjects.

## Methods

### Subjects

This study was approved by the Institutional Review Board of the Joint Shantou International Eye Center (JSIEC), Shantou University and the Chinese University of Hong Kong. All eligible subjects received an explanation of the study and signed an informed consent form in accordance with the principles embodied in the Declaration of Helsinki. A total of 228 normal subjects and 93 primary open-angle glaucoma (POAG) patients were enrolled in the study. All subjects were examined at JSIEC between August 2013 and March 2014.

Detailed medical histories were taken from all subjects. The patients underwent comprehensive ophthalmic examinations, including best corrected visual acuity, intraocular pressure, refractive error, slit-lamp biomicroscopy, fundus examination, visual field (VF) evaluation and spectral domain-optical coherence tomography (SD-OCT) scan. Humphrey SITA standard 24–2 visual field testing was used, and only subjects with the fixation losses <20% and false-positive and false-negative responses <15% were enrolled.

Inclusion criteria for normal subjects were as follows: best-corrected visual acuity (BCVA) ≥0.5, intraocular pressure ≤21 mmHg, spherical refraction between -6.0 and 6.0 diopters (D), normal optic disc appearance, normal visual field, and the absence of other ocular diseases, diabetes, and neurological disorders that may influence VF results. The eye was randomly chosen if both eyes were eligible.

The POAG patients were included if all the following criteria were met: elevated intraocular pressure (IOP) >21 mmHg on at least two separate visits; glaucomatous optic disc appearance; VF damage to two or more contiguous points with a pattern deviation sensitivity loss of P<0.01 or three or more contiguous points with a sensitivity loss of P<0.05 in the superior or inferior arcuate areas, or a 10-dB difference across the nasal horizontal midline at two or more adjacent locations and an abnormal result on the glaucoma hemifield test; wide and open angle on gonioscopy; no other obvious causes for these changes; glaucomatous optic disc appearance of the neuroretinal rim; and asymmetry of the cup disc ratio ≥0.2 between two eyes without asymmetric refraction.

The raw data were obtained from the Topcon 3D-OCT 2000 (software version: 8.11.003.04), and macula-centered SD-OCT volumes were acquired. Each SD-OCT volume was 512×128×885 voxels or 6×6×2.3 mm^3^ in physical dimensions. The SD-OCT volumes with image quality lower than 45 were excluded (Image quality score was provided by the onboard OCT software. This is a quantitative parameter representing the signal strength of the scanned multi-frame image. The value is automatically obtained when collecting the data.).

### 3D-OCT image analysis

**Fig 1:**
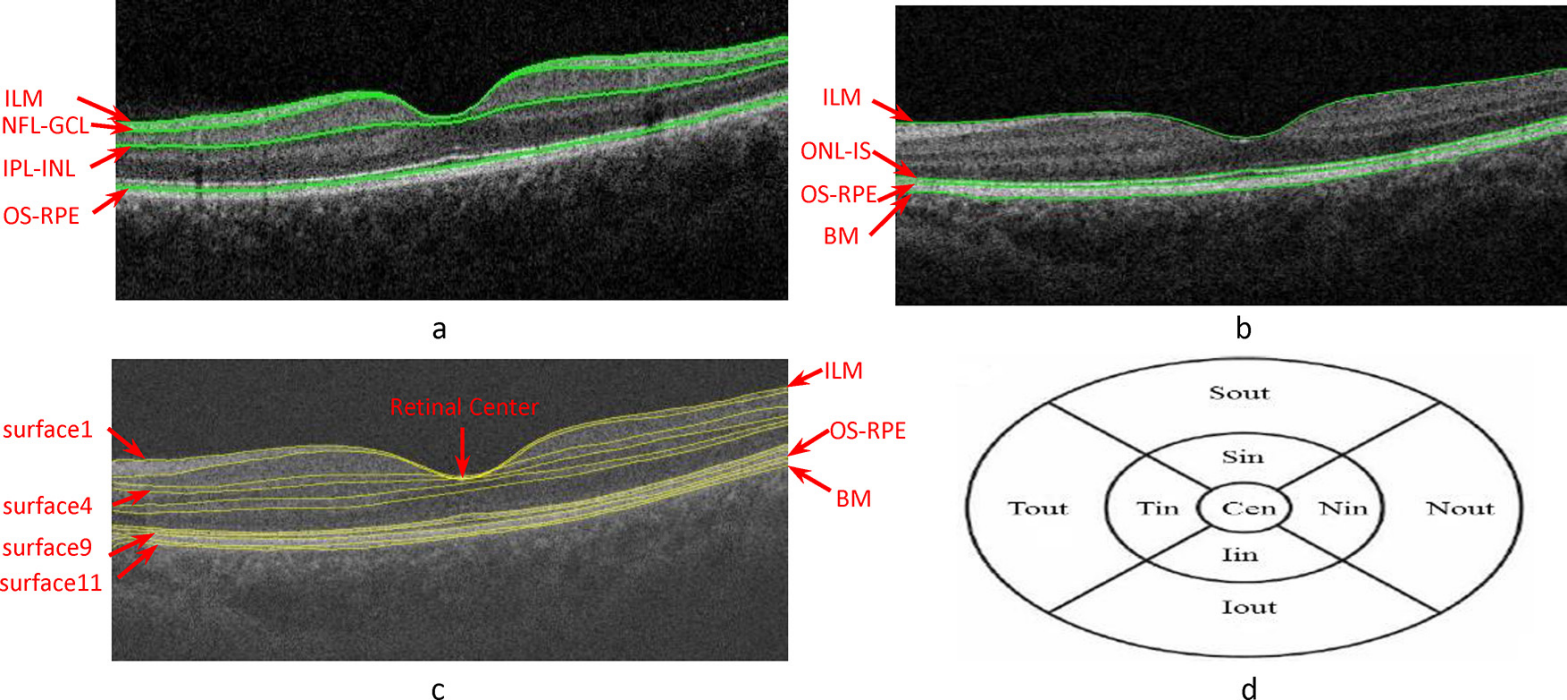
Computation of macular GCC thickness and macular thickness in 9 sectors on the ETDRS chart. (a) Macular B-scan of the following intraretinal surfaces detected by the Topcon algorithm: inner limiting membrane (ILM), NFL-GCL, IPL-INL, and OS-RPE. (b) Macular B-scan of the following intraretinal surfaces detected by Topcon algorithm: ILM, ONL-IS, OS-RPE and Bruch’s membrane (BM). (c) Macular B-scan of the following intraretinal surfaces identified using our graph-based algorithm: ILM, NFL-GCL, GCL-IPL, IPL-INL, INL-OPL, OPL-ONL, ONL-IS, IS-OS, OS-RPE, Verhoeff’s membrane (VM), and RPE/BM. (d) ETDRS scheme applied for the measurement of retinal thickness. The area of Cen represents the fovea. Mean macular thickness was calculated for the superior (S), inferior (I), temporal (T), and nasal (N) areas with diameters of 1 mm, 3 mm, and 6 mm.

Eleven surfaces of the 3D-OCT volumetric macula-centered scan were segmented using a graph search algorithm, which is a fast, three-dimension, automatic graph-theoretical segmentation approach (Fig 1(C)). The graph search approach employed here transforms the intraretinal layer segmentation problem into an optimal surface problem [21–26]. The workflow includes two parts: a preprocessing step and layer segmentation step. During the preprocessing step, a curvature anisotropic diffusion filter was used to reduce the OCT speckle noise. Subsequently, the graph search based Iowa algorithm[23, 27] of retinal layer segmentation is applied to segment retinal layer. First, four multi-scale OCT volumes were created by subsampling by a factor of 2 in the z-axis. Then the gradient magnitude in z direction was calculated as a cost function. During the layer segmentation part, a weighted directed graph G = (v, e) was constructed, which was composes of a node set V and an arc set E. In the graph, the nodes v∈V corresponded to image voxels, and arcs < v_i_, v_j_>∈E connected the nodes v_i_, v_j_. The cost (or weight) of the node v∈V was derived from the cost function and can be expressed as some measure (e.g., gradients) of the corresponding voxels belonging to the surface[27].By finding an optimal closed set in a vertex-weighted graph, the approach was able to segment the intraretinal surfaces. At higher resolutions, the surfaces were detected near the locations obtained from the next lower resolution.

The commercially available Topcon in-built algorithm (Topcon 3D-OCT 2000, 8.11.003.04) is a two-dimension approach that determines intraretinal surfaces with a slice-by-slice approach, although the algorithms have not been published. Only 6 intraretinal surfaces (ILM, NFL, IPL, IS/OS, RPE, BM) can be obtained using the Macular B-scans.

### Thickness of the separate layers

The intraretinal layer thickness of the RNFL, RGCL+IPL, and GCC (RNFL+RGCL+IPL) were obtained from the Topcon in-built algorithm.

Because 11 intraretinal surfaces can be detected using the proposed 3D graph-based algorithm, we can calculate the thickness of any layer or several layers. In this study, the intraretinal layer thickness of the RNFL, RGCL+IPL, and GCC were calculated utilizing the z-axis value of surface 1 (ILM) to surface 4 (IPL-INL).

### Retinal thickness of the 9 sectors of the ETDRS chart

In this step, we first found the lowest location of the first surface (ILM) in the image, which is used as a center point of the Early Treatment Diabetic Retinopathy (ETDRS) chart [28]. The ETDRS plot includes three circles that divide the macula into two rings: the inner circle of the ETDRS plot with a diameter of 1 mm is Cen region, the middle circle of the ETDRS plot with a diameter of 3 mm, and the outer circle of the ETDRS plot with a diameter of 6 mm. These circles are centered on the fovea. Subsequently, the macula is further divided into four quadrants: Tout and Tin correspond to the outer and inner temporal quadrants, Nout and Nin correspond to the outer and inner nasal quadrants, Sout and Sin correspond to the outer and inner superior quadrants, and areas Iout and Iin correspond to the outer and inner inferior quadrants (Fig 1(D)). Finally, the information of surface 1 (ILM) and surface 9 (inner surface of the RPE) are utilized, and the thickness of every 9 sectors and the overall circular region on the ETDRS chart of the macula-centered retina is measured.

For Topcon, the retinal thicknesses (from the ILM to the inner surface of the RPE) of the 9 sectors on the ETDRS Chart were obtained from the Topcon in-built algorithm.

### Statistical analysis

The mean thickness of the RNFL, GCL+IPL, and RNFL+GCL+IPL and the macular-central retinal thickness of the 9 sectors of the ETDRS chart calculated using the two different algorithms were analyzed with Pearson’s correlation. Correlation coefficients (r) and mean differences were calculated. Bland-Altman plots were also assessed to further analyze the agreement between the Topcon algorithm and our algorithms. The software SPSS (Version 16.0, IBM) and MedCalc (http://www.medcalc.org/) were used to conduct the statistical analysis.

## Result

The average age and image quality score of the 228 normal subjects and 93 glaucoma patients was 46.9±16.9 years and 50.0±16.1 and 57.3±4.5 years and 54.5±4.3, respectively. There was no significant difference with respect to age or image quality score between the two groups. The study population characteristics are summarized in Table 1.

**Table 1:** Characteristics of the Included Subjects.

	Normal	Glaucoma	P-value
Sex(male/female)	102/126	57/36	0.005
Age(years)	46.9±16.9	50.0±16.1	0.129
SE refraction(D)	-0.7±1.9	-1.0±2.5	0.869
Axial Length(mm)	23.7±1.2	24.2±1.4	0.634
Image Quality	58.1±4.5	54.9±4.8	<0.001
MD(dB)	-1.3±1.3	-10.6±8.6	<0.001
PSD(dB)	1.6±0.6	6.9±4.1	<0.001

The mean macular layer thickness of the RNFL, GCL+IPL, and GCC (RNFL+GCL+IPL) as measured by the Topcon algorithm and our graph-based algorithm were as follows: normal subjects (36.5 μm, 69.3 μm, and 105.8 μm vs 37. 7 μm, 71.3 μm, and 108.9 μm) and glaucoma subjects (26.7 μm, 61.2 μm, and 87.0 μm vs 30.4 μm, 59.2 μm, and 89.5 μm) (Table 2). The macular thickness of the 9 sectors of the ETDRS obtained by the Topcon algorithm and the graph-based algorithm are also compared in Table 3.

**Table 2:** Comparison of Macular GCC^*^ Thickness Measured by the Topcon Algorithm and the Graph-Based Algorithm (Mean ± SD).

		Normal subjects					Glaucoma subjects			
	Topcon	Graph-Based	Correlation coefficient	Mean Difference (Topcon-Graph Based)	p-value	Topcon	Graph-Based	Correlation coefficient	Mean Difference (Topcon-Graph Based)	p-value
RNFL	36.5±4.2	37.7±4.3	0.86	-1.1±1.8	<0.01	26.7±9.5	30.4±6.4	0.78	-3.7±6.4	<0.01
GCL+IPL	69.3±5.1	71.3±4.8	0.91	-2.0±2.0	<0.01	61.2±7.5	59.2±11.2	0.83	2.0±6.7	<0.01
RNFL+GCL+IPL	105.8±7.7	108.9±7.3	0.90	-3.1±2.6	<0.01	87.0±16.2	89.5±16.4	0.90	-2.5±7.6	<0.01

*GCC: ganglion cell complex.

**Table 3:** Comparison of the Macular Thickness of 9 Sectors on ETDRS Measured by the Topcon Algorithm and the Graph-Based Algorithm (Mean ± SD).

	Normal subjects								Glaucoma subjects			
	Topcon	Graph-Based		Correlation coefficient		Mean Difference (Topcon-Graph Based)	p-value	Topcon	Graph-Based	Correlation coefficient	Mean Difference (Topcon-Graph Based)	p-value
Cen	223.6±18.5		217.3±17.8		0.95	6.3±5.9	<0.01	225.1±21.6	216.8±20.0	0.84	8.3±11.8	<0.01
Sin	299.7±17.0		296.5±17.2		0.96	3.2±4.5	<0.01	284.7±22.4	281.7±22.7	0.93	3.0±8.7	<0.01
Nin	300.9±18.0		296.7±17.4		0.93	4.2±6.5	<0.01	285.2±23.2	283.0±23.8	0.90	2.2±11.0	>0.06
Iin	295.7±17.0		293.7±16.4		0.94	2.1±5.7	<0.01	276.5±25.1	275.2±24.3	0.95	1.3±7.8	>0.12
Tin	284.0±16.5		283.6±16.3		0.95	0.4±5.3	>0.17	269.1±21.3	267.0±21.4	0.91	2.1±8.9	>0.02
Sout	265.7±15.0		262.8±15.5		0.94	2.8±5.5	<0.01	246.5±20.3	245.3±20.1	0.93	1.2±7.8	>0.12
Nout	281.8±16.6		284.3±21.3		0.86	-2.5±11.4	<0.01	263.2±22.9	263.4±26.2	0.71	-0.2±19.0	>0.90
Iout	254.0±14.2		251.6±15.2		0.90	2.5±6.7	<0.01	235.5±21.2	232.1±20.8	0.92	3.4±5.6	<0.01
Tout	249.7±14.5		246.2±15.4		0.79	3.6±9.7	<0.01	234.3±19.1	234.9±22.1	0.70	-0.6±16.2	>0.70
Ave	268.9±13.5		267.0±14.1		0.96	1.9±4.0	<0.01	251.4±18.6	250.7±19.0	0.93	1.0±7.4	>0.20

**Fig 2:**
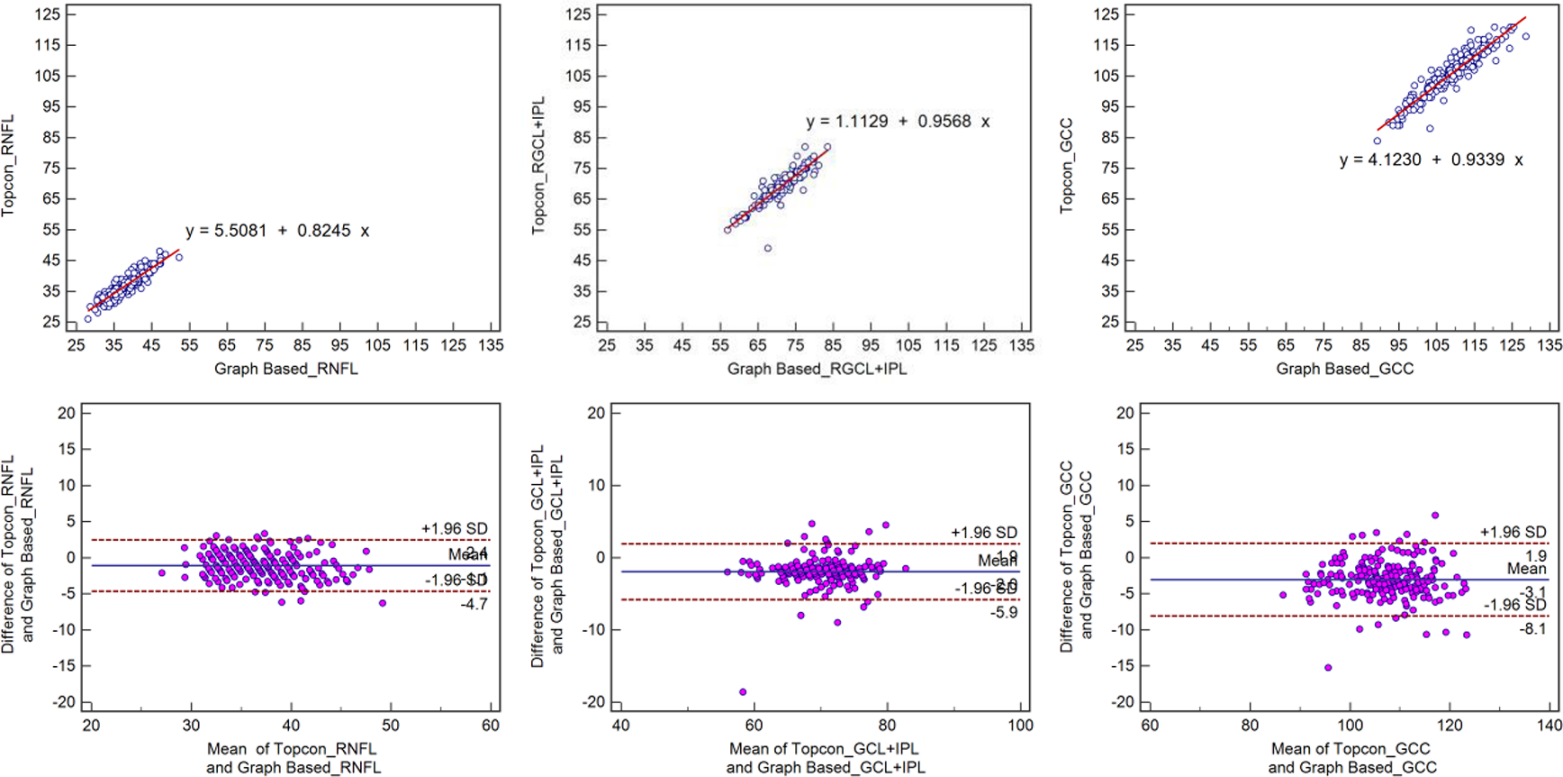
Scatter plots and Bland-Altman plot for normal subjects. **First row:** Scatter plots demonstrating the correlation between the Topcon algorithm and the graph-based algorithm with respect to retinal GCC thickness. **Second row:** Bland-Altman plot demonstrating the agreement between the Topcon algorithm and the graph-based algorithm with respect to retinal GCC thickness.

**Fig 3:**
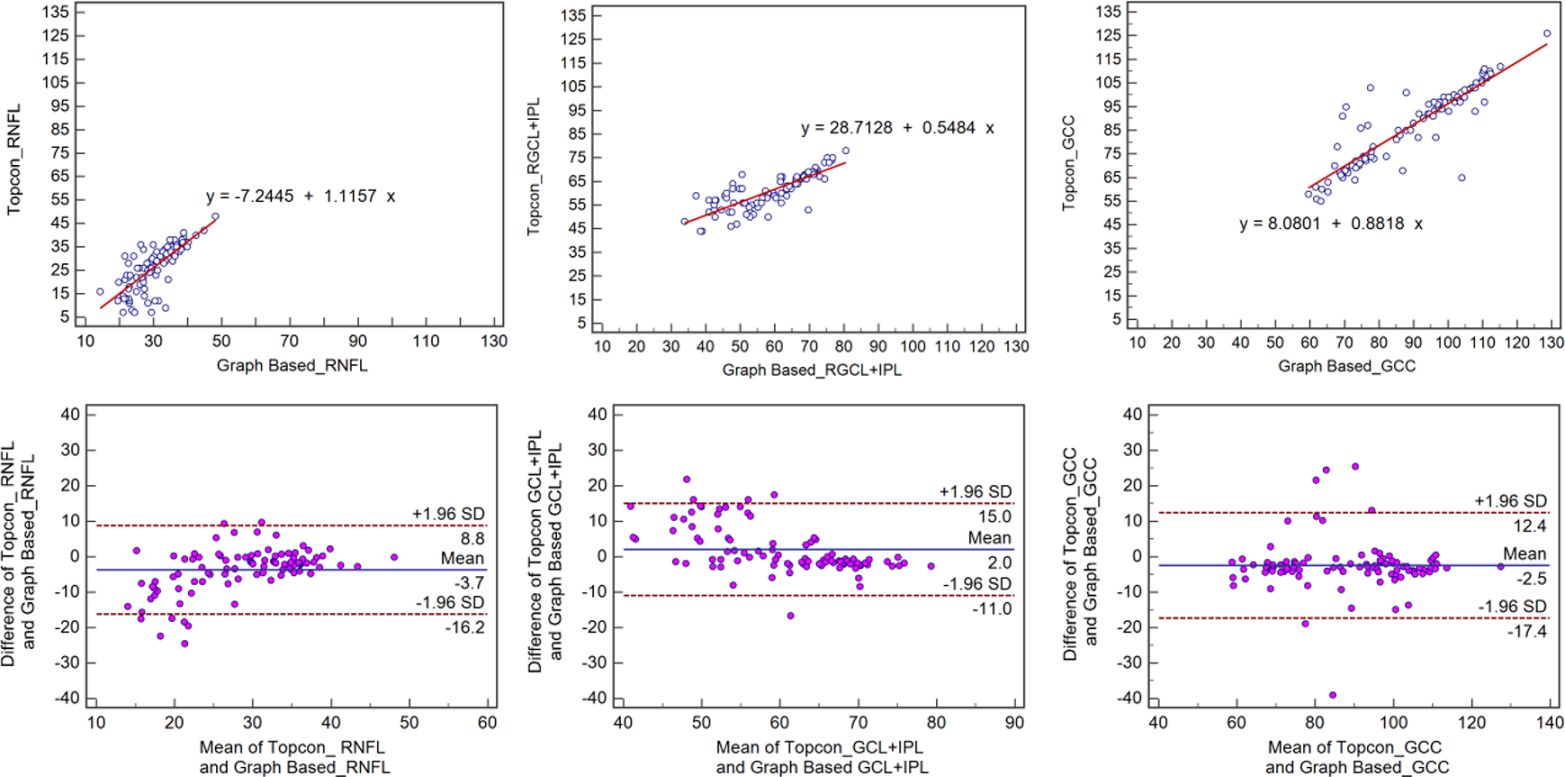
Scatter plots and Bland-Altman plot for glaucoma subjects. **First row:** Scatter plots demonstrating the correlation between the Topcon algorithm and the graph-based algorithm with respect to retinal GCC thickness. **Second row:** Bland-Altman plot demonstrating the agreement between the Topcon algorithm and the graph-based algorithm with respect to retinal GCC thickness.

A high degree of correlation was observed between the results obtained from the two methods as evidenced in Table 2 and Table 3, first row of Figs 2 and 3, with r>0.78. 

**Fig 4:**
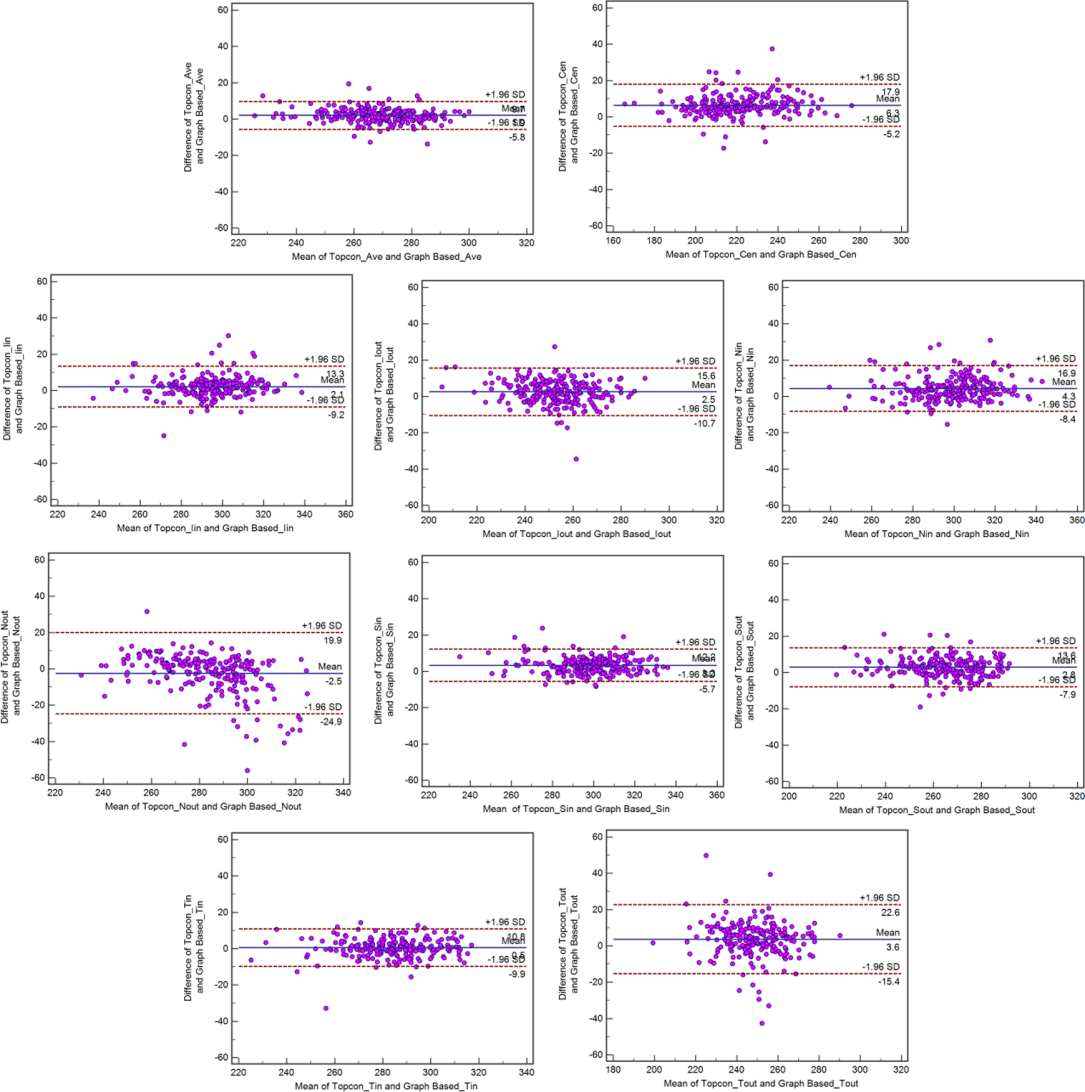
Bland-Altman plot demonstrating the agreement between the Topcon algorithm and the graph-based algorithm with respect to the retinal thickness of 9 sectors on ETDRS for normal subjects.

**Fig 5:**
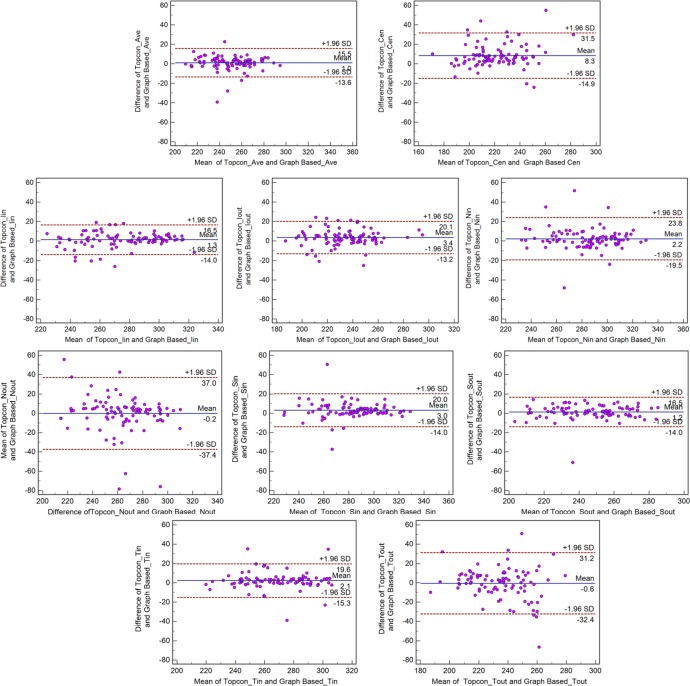
Bland-Altman plot demonstrating the agreement between the Topcon algorithm and the graph-based algorithm with respect to retinal thickness of 9 sectors on ETDRS for glaucoma subjects.

Bland-Altman plots demonstrated significant agreement with respect to the measures obtained with the two methods (Figs 2–5). The mean differences in the RNFL, GCL+IPL, and GCC (RNFL+GCL+IPL) thickness measurements obtained using the two methods were -1.1 μm, -2.0 μm, and -3.1 μm for the normal subjects and -3.7 μm, 2.0 μm, and -2.5 μm for the glaucoma subjects, respectively. The span of 95% limits of agreement ranged between 7.1 μm and 10.0 μm for normal subjects and between 22.7 μm and 28.0 μm for glaucoma subjects.

The mean differences in the sectoral macular thickness were <4.3 μm for both normal and glaucoma subjects. However, the mean differences in the Cen region were 6.3 μm for normal subjects and 8.3 μm for glaucoma subjects. The span of 95% limits of agreement ranged between 15.5 μm (Ave) and 44.8 μm (Nout) for normal subjects and between 29.1 μm (Ave) and 74.4 μm (Nout) for glaucoma subjects.

## Discussion

Many studies have compared retinal thickness measurements obtained by different commercial OCT machines.[15, 18–20]. Leung et al. studied 35 healthy subjects and reported that the span of 95% limits of agreement between the Carl Zeiss Stratus OCT and Topcon 3D OCT was 33.9 μm for foveal thickness and 21.3 μm for total macular thicknesses. In the study by Sánchez-Dalmau et al. the authors compared three different OCT devices with respect to the estimated RNFL thickness in 50 eyes with neuro-ophthalmological disorders of the afferent visual pathway. The authors reported that the agreement between Stratus and Cirrus was high, although there was only poor agreement between the 3D OCT-1000 and the Stratus or Cirrus. These reports used different images created by different machines at different times; therefore, it is almost impossible to measure and compare the performance of each image analysis algorithm. The present study used the Topcon 3D OCT image as a reference and analyzed thicknesses using the Topcon algorithm and our graph-based algorithm.

In this study, we compared the measurements of the retinal macular GCC thickness and the retinal thickness of 9 sectors of the ETDRS chart calculated according to the latest Topcon 3D-OCT 2000 built-in algorithm and our graph-based algorithm in both normal and glaucoma subjects.

Our graph-based algorithm performed similarly to the Topcon manufacturer-supplied algorithm. There was a strong correlation and a high degree of agreement between these two algorithms with respect to measure macular thicknesses in the subjects with and without glaucoma. However, our algorithm can segment the retina into more layers than the Topcon in-built algorithm and can therefore provide more information with respect to layer thickness for the diagnosis and treatment of glaucoma.

With respect to retinal macular GCC thickness measurements, the two algorithms exhibited a strong correlation in both normal and glaucoma subjects, with the exception of the RNFL value (0.86 for normal and 0.78 for glaucoma). The reason for this difference may be related to the thickness of the retinal layer; thinner layers may correlate with a greater variation in the segmentation of layers. Furthermore, the mean difference of the RNFL was higher in glaucoma subjects but lower in normal subjects (shown in Table 2). This finding may be attributable to the morphologic changes caused by glaucoma that affect the result of the retinal layers segmentation, particularly resulting in significant thinning of the RNFL and GCL. Therefore, layer segmentation inaccuracies may be observed, and the situation may be more serious in glaucoma. The slightly lower correlation may be caused by the segment error.

**Fig 6:**
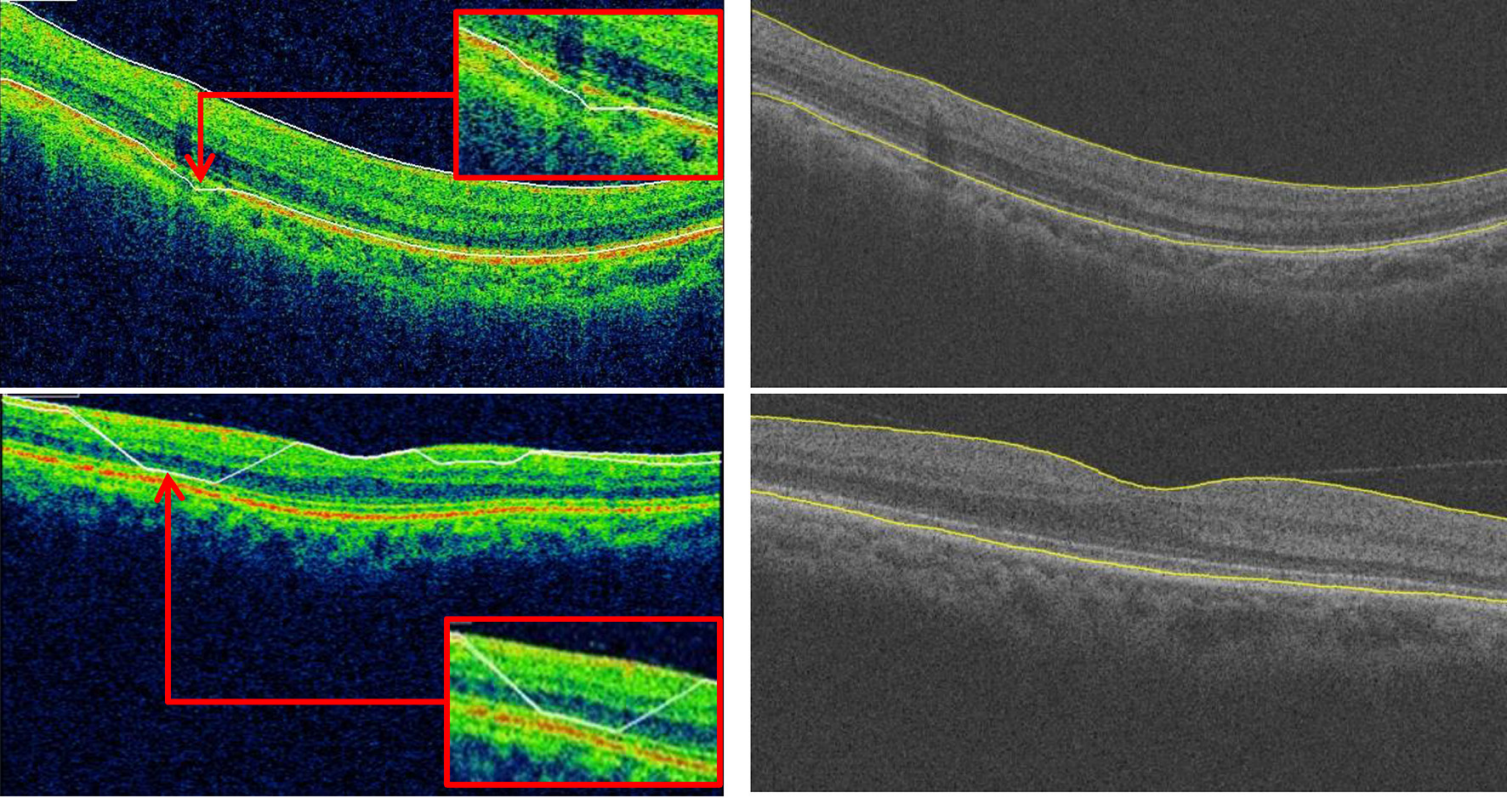
Comparison of segmentation results. Left, Topcon result. Right, our result.

With respect to the macular thickness of the 9 sectors of the ETDRS, we also observed a strong correlation between the Topcon built-in algorithm and the graph-search algorithm, However, there were some exceptions; in Nout and Tout, the correlation between the two methods was less than in other subfields in both normal and glaucoma subjects. Although the technological details of the Topcon algorithm are not available, after examining all data, there were obvious layer segmentation inaccuracies in the Topcon algorithm (Fig 6). The Topcon algorithm cannot overcome local blurry image information, such as local signal dropout and vessel shadows, which can cause local layer segmentation inaccuracies, especially for the surface of the RPE layer on the outer annulus of the ETDRS chart. However, our graph-based algorithm uses all 3D information when identifying and segmenting the retinal layers and is therefore able to overcome this problem much better.

**Fig 7:**
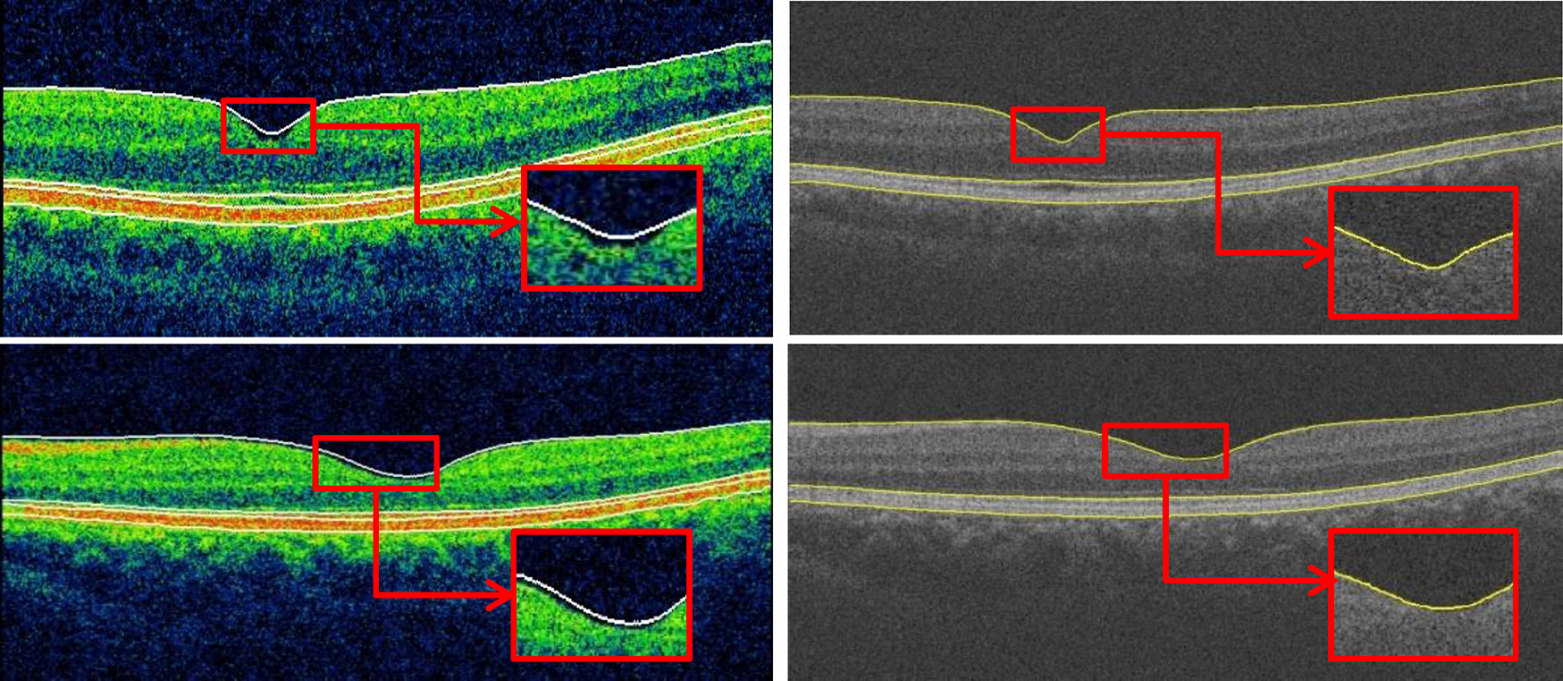
Comparison of segmentation results in macular region. Left, Topcon result. Right, our result.

Bland-Altman analysis revealed good agreement between the Topcon built-in algorithm and our graph-based algorithm with respect to the macular thickness measurements. As shown in the Table 3, although the thickness difference between the Topcon algorithm and our graph-based algorithm was low with respect to the macular GCC thickness measurement (<3.2 μm), the macular thickness was larger in the Cen sector (6.3 μm for normal subjects and 8.3 μm for glaucoma subjects) compared with the other sectors (<4.3 μm). In examining the all data, we observed that this difference is due to the fact that the ILM was segmented by the Topcon algorithm in a position higher than actual position in the fovea in a number of cases (Fig 7).The spans of 95% limits of agreement were narrow for all macular thickness measurements, but these limits were broader in glaucoma subjects compared with normal subjects, particularly with respect to the Nout region (Figs 2–5). As seen in the Bland-Altman plots, some plots exhibited the obvious differences between the two methods (Figs 4 and 5). After visual examination of the boundaries segmented by the Topcon algorithm and our graph-based algorithm, we observed obvious segmentation inaccuracies in these Topcon cases but not in our cases (Figs 6 and 7). We have visually checked all B-scan for all layers across the 321 macular volumes, and find we have better segmentation results in macular region (1mm x 1mm) than Topcon algorithm do (Fig 7). It is also possible that the Topcon’s algorithm cannot segment the OCT volumes correctly if there are motion artifacts or rotational differences, which are more seriously in glaucoma subjects. However, our true 3D graph-based algorithm segments the layer successfully.

In this study, as without the manual segmentation performed by the ophthalmologist, we did not quantitatively analyze the layer segmentation performance of our algorithm. In the future, we will compare the automatic segmentation results with the manual segmentation results and quantitatively analyze our algorithm performance.

The Topcon 2000 software were installed on a Windows 7 workstation with 3.4GHz CPU and 4G RAM, for the normal dataset(volume was 512×128×885 voxels), the computation time for layer segmentation of 4 surfaces is about 30 seconds; while for the proposed algorithm running on a Windows 7 workstation with 3.4GHz CPU and 4G RAM, for the same size normal dataset (volume was 512×128×885 voxels), the computation time for layer segmentation of 11 surfaces is also about 30 seconds. However, we can get 11 surfaces segmentation results while Topcon can get only 4 surfaces.

In summary, our graph-based algorithm has good performance with respect to segmentation and measurement in both normal and glaucoma subjects, and has exhibited a high degree of agreement with the Topcon algorithm. Moreover, our graph-based algorithm can segment the retina into more layers than the Topcon built-in algorithm do. So the proposed method can provide more information to the clinician in the process of clinician judgment.
